# High-concentration hydrogen protects mouse heart against ischemia/reperfusion injury through activation of thePI3K/Akt1 pathway

**DOI:** 10.1038/s41598-017-14072-x

**Published:** 2017-11-01

**Authors:** Ouyang Chen, Zhiyong Cao, He Li, Zhouheng Ye, Rongjia Zhang, Ning Zhang, Junlong Huang, Ting Zhang, Liping Wang, Ling Han, Wenwu Liu, Xuejun Sun

**Affiliations:** 1 Department of Navy Aviation Medicine, Faculty of Naval Medicine, Second Military Medical University, Shanghai, 200433 People’s Republic of China; 2 Department of Clinical Medicine, Second Military Medical University, Shanghai, 200433 People’s Republic of China; 3 Department of Diving Medicine, Faculty of Naval Medicine, Second Military Medical University, Shanghai, 200433 People’s Republic of China; 4 Central Laboratory, Faculty of Naval Medicine, Second Military Medical University, Shanghai, 200433 People’s Republic of China; 5 Department of Cardiology, No.411 Hospital of PLA, Shanghai, 200081 People’s Republic of China; 6 Department of Anesthesiology, Fuzhou General Hospital of PLA, Fuzhou, 350025 Fujian Province People’s Republic of China

## Abstract

The study investigated the role of Akt1 through the cardioprotection of high-concentration hydrogen (HCH). C57BL/6 mice were randomly divided into the following groups: sham, I/R, I/R + HCH, I/R + HCH + LY294002 (PI3K inhibitor), I/R + HCH + wortmannin (PI3K inhibitor), I/R + LY294002, and I/R + wortmannin. After 45 min of ischemia, HCH (67% H_2_ and 33% O_2_) was administered to mice during a 90-min reperfusion. To investigate the role of Akt1 in the protective effects of HCH, mice were divided into the following groups: I/R + A-674563 (Akt1 selective inhibitor), I/R + HCH + A-674563, I/R + CCT128930 (Akt2 selective inhibitor), and I/R + HCH + CCT128930. After a 4-h reperfusion, serum biochemistry, histological, western blotting, and immunohistochemical analyses were performed to evaluate the role of the PI3K-Akt1 pathway in the protection of HCH. *In vitro*, 75% hydrogen was administered to cardiomyocytes during 4 h of reoxygenation after 3-h hypoxia. Several analyses were performed to evaluate the role of the Akt1 in the protective effects of hydrogen. HCH resulted in the phosphorylation of Akt1 but not Akt2, and Akt1 inhibition markedly abolished HCH-induced cardioprotection. Our findings reveal that HCH may exert cardioprotective effects through a PI3K-Akt1-dependent mechanism.

## Introduction

Cardiovascular disease (CVD) is one of the leading causes of death worldwide. According to the latest statistics, more than 2,200 Americans die of CVD each day, which is equivalent to an average of one death every second^1^. Acute myocardial infarction (AMI) is a potentially fatal event. Currently, restoring blood flow is the most effective and important therapy for AMI. However, recanalization may cause myocardial ischemia/reperfusion (I/R) injury, in which a variety of cytotoxic cascades are activated, leading to cardiomyocyte apoptosis and inflammation^2–4^.

Phosphatidylinositol-4,5-bisphosphate 3-kinases (PI3Ks), which lie upstream of Akt, are a family of evolutionarily conserved lipid kinases that mediate many cellular responses in both physiological and pathophysiological states^5^. The PI3K/Akt signaling pathway is one of the most important signal transduction pathways related to the survival and functions of cardiomyocytes^6^. Akt, a serine/threonine kinase, is at the center of the signaling pathways that regulate many cellular functions, including growth, survival, and metabolism^7^. Activation of Akt promotes cardiomyocyte survival by regulating various downstream proteins, including glycogen synthase kinase-3β (GSK-3β) and Bcl-2-associated death promoter (BAD), to inhibit caspase-3 activation and subsequent cell apoptosis^8^. Additionally, Akt may inhibit nuclear factor (NF)-κB activation, leading to the suppression of inflammation. Activated Akt also inhibits c-Jun N-terminal kinase (JNK) to ameliorate cell necrosis. p38 mitogen-activated protein kinase (p38 MAPK) is an upstream trigger of Akt phosphorylation, which interferes with the processes leading to cell necrosis^6^.

The Akt subfamily comprises three mammalian isoforms (Akt1, Akt2, and Akt3), which are encoded by distinct genes located on different chromosomes and share more than 80% amino acid identity^9^. Despite these structural similarities, knockdown of a specific Akt isoform in mice leads to different phenotypes ascribed to non-redundant functions to each of these isoforms^10–16^. Akt1 is ubiquitously expressed in all tissues, while Akt2 presents predominantly in insulin-responsive tissues and Akt3 is mainly expressed in the brain^12,15,17–19^. In the heart, Akt1 and Akt2 have high expression, and functionally, Akt1 is essential for promoting cell survival^11,12^ and cardiac growth^20^, while Akt2 is implicated in maintaining glucose homeostasis^21^.

In 2007, Ohsawa *et al*. reported that hydrogen gas (H_2_) inhalation could protect the brain against I/R injury by selectively neutralizing hydroxyl radicals and peroxynitrite^22^. Since then, the protective effects of H_2_ have been studied extensively in multiple organs. Compared with classical antioxidants, H_2_ is a small molecule that can easily dissipate throughout cells. Although the cardioprotective effects of H_2_ have been confirmed in previous studies^23,24^, the concentration of H_2_ is usually no more than 4% due to safety concerns, and the specific mechanism underlying the cardioprotective effects of H_2_ remains poorly understood. Recently, our group treated various diseases with high concentrations of hydrogen (HCH) gas (67% H_2_ and 33% O_2_) in animal models, and its protective effects were confirmed^25,26^. This mixed gas is produced using an AMS-H-01 hydrogen/oxygen nebulizer (Asclepius, Shanghai, China), which can produce H_2_ and O_2_ by electrolyzing water. However, it remains unknown whether HCH is also protective towards myocardial I/R injury and, if so, the mechanism underlying these potential effects is unclear. In the present study, we investigated whether HCH could exert cardioprotective effects on I/R injury *in vivo* and *in vitro*, and whether the PI3K/Akt pathway was involved in these effects.

## Results

### Hydrogen concentration of the blood and myocardium significantly increased after HCH treatment

The H_2_ concentration was measured in the arterial and venous blood of mice in the sham, I/R, and H_2_ groups after HCH treatment. As shown in Supplementary Fig. 1A, the H_2_ concentration increased in both the arteries and the veins of mice after inhalation of HCH. The venous H_2_ concentration was approximately 50% of the arterial H_2_ concentration. In addition, there was no significant difference in the arterial and venous H_2_ concentrations between the sham group and the I/R group (*P* > *0.05*). In addition, inhalation of H_2_ did not affect the blood pH value (7.38 ± 0.05; data not shown).

The H2 concentration of the myocardium was also measured after a 90-min HCH treatment. The H2 concentration was significantly different among the sham (3.93 ± 1.85 μL/kg), I/R (5.25 ± 1.36 μL/kg), and I/R + HCH (144.01 ± 14.14 μL/kg) groups (Supplementary Fig. 1B). The H2 concentration in H2-treated mice was significantly higher than those in the sham and I/R groups (*P* < 0.05), but there was no significant difference between the sham group and the I/R group (*P* > 0.05).

### HCH improved I/R-induced cardiac injury, which was attenuated by PI3K/Akt inhibitors

**Figure 1:**
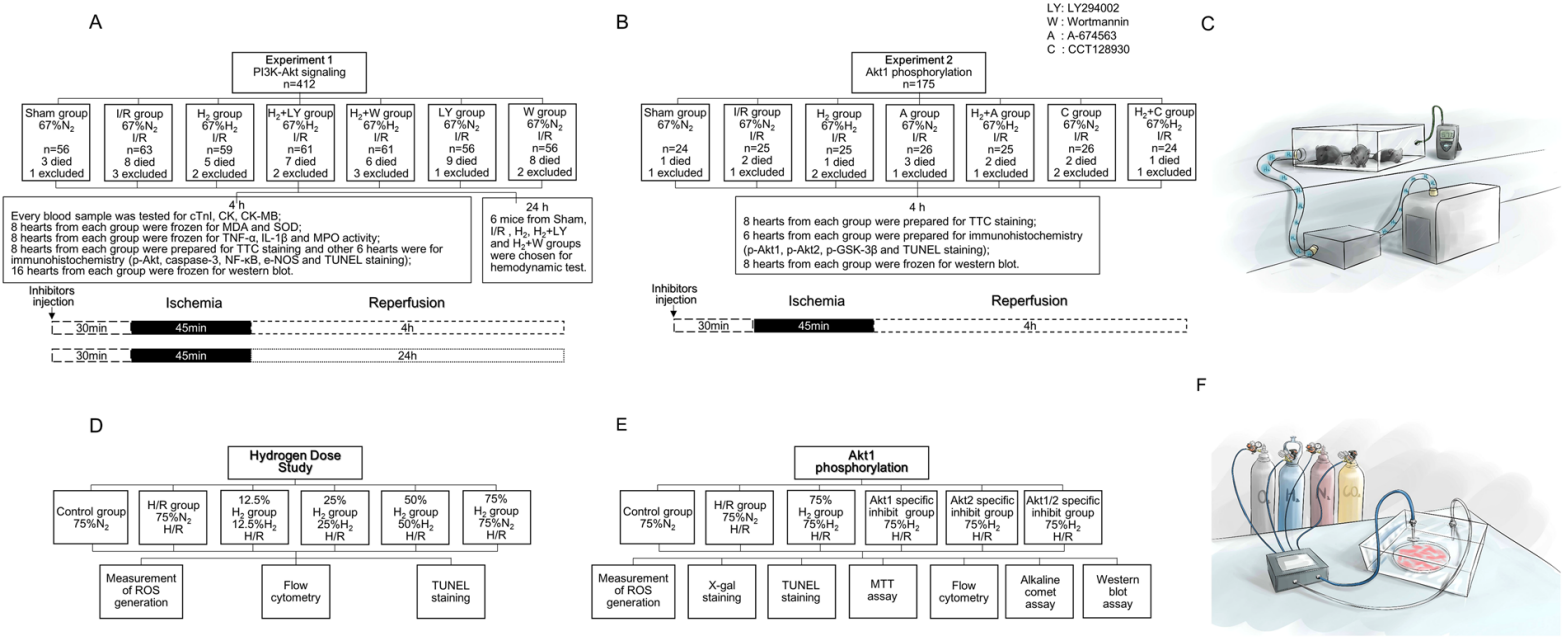
Flow chart of the study. The study included *in vivo* animal experiments and *in vitro* experiments. In experiment 1, mice were divided into seven groups: sham, I/R, I/R + H_2_, I/R + H_2_ + LY, I/R + H_2_ + W, I/R + LY, and I/R + W. A 90-min inhalation of 66.7% H_2_ and 33.3% O_2_ gas was initiated immediately after 45 min of ischemia. Cardiac enzymes, infarct area, oxidative parameters, inflammatory parameters, apoptotic parameters, and the phosphorylation of Akt-related proteins were measured 4 h after reperfusion. A hemodynamic test was performed 24 h after reperfusion. In experiment 2, mice were divided into seven groups: sham, I/R, I/R + H_2_, I/R + A, I/R + H_2_ + A, I/R + C, and I/R + H_2_ + C. Cardiac enzymes, apoptotic parameters, and the phosphorylation of Akt-related proteins were measured 4 h after reperfusion. In the *in vitro* experiments, flow cytometry, TUNEL staining, and measurement of ROS generation were conducted after 4 h of reoxygenation to confirm the optimal dose of H_2_. Cardiomyocytes were assigned randomly into six groups for investigation of the role of Akt1 and Akt2 in the protective effects of 75% H_2_ as follows: control, H/R, H/R + HCH, H/R + A + HCH, H/R + C + HCH, and H/R + A + C + HCH. Measurement of ROS generation, X-gal staining, TUNEL staining, MTT assay, flow cytometry, alkaline comet assay, and western blot were performed after 4 h of reoxygenation.

**Figure 2:**
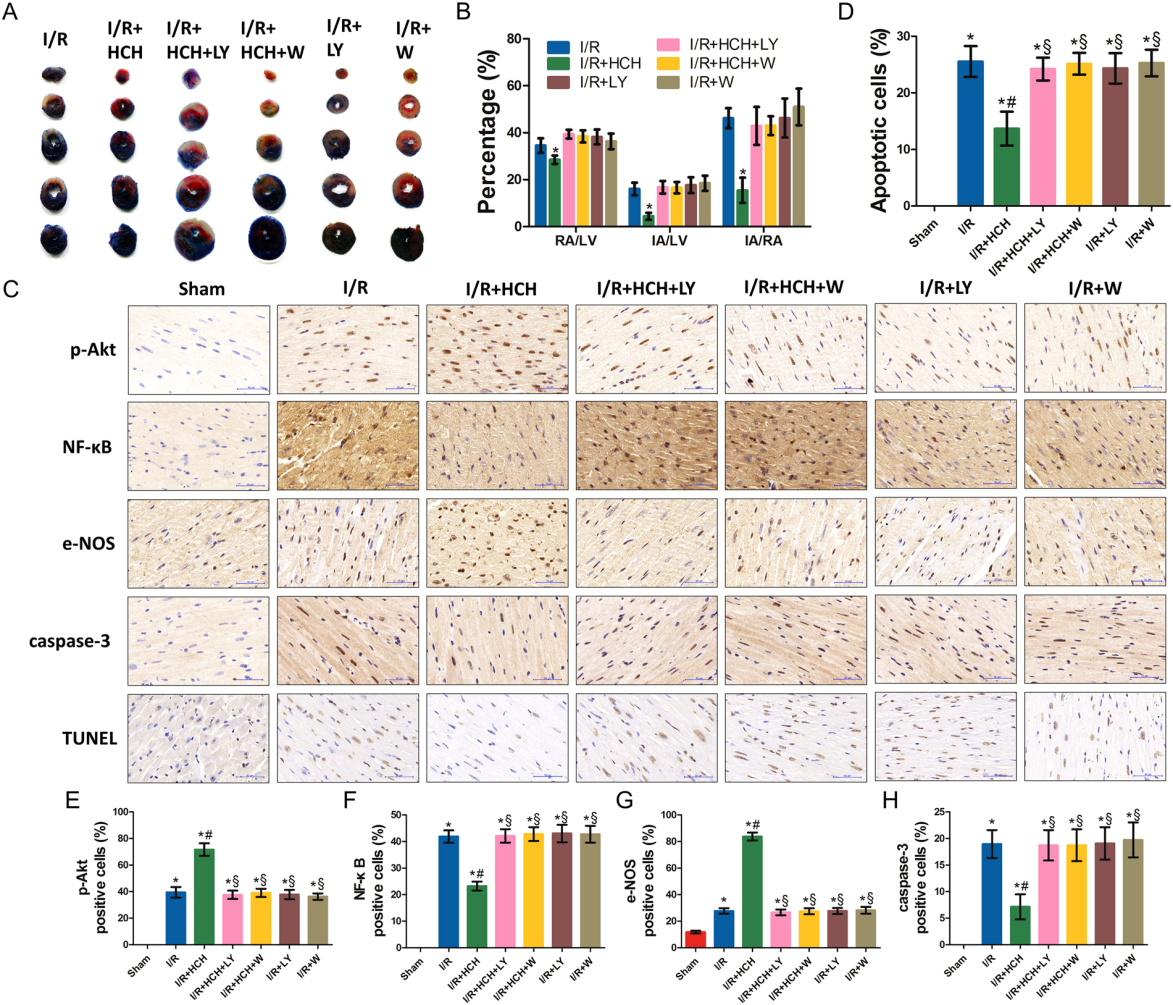
Morphological examination from experiment 1. Four hours after reperfusion, mice were sacrificed and the hearts were collected for determination of the infarct area (n = 56) and immunohistochemistry (n = 42). HCH significantly reduced the infarct area following I/R (*P* < 0.05). There were no significant differences in RA/LV, IA/RA, and IA/LV among the I/R and inhibitor groups (I/R + HCH + LY294002, I/R + HCH + wortmannin, I/R + LY294002, and I/R + wortmannin) (*P* > 0.05) (**A**,**B**). Immunohistochemistry was performed for p-Akt, NF-κB, e-NOS, and caspase-3. Commercially available kits were used for TUNEL staining (**C**). HCH significantly increased the number of cells positive for p-Akt and e-NOS and significantly decreased the number of cells positive for NF-κB, caspase-3, and TUNEL. PI3K inhibitor treatment abolished the protective effects of HCH. *P < 0.05 vs. sham; ^#^ *P* < 0.05 vs. I/R; ^§^ *P* < 0.05 vs. I/R + HCH. Magnification = ×400.

We first investigated the cardioprotective effects of HCH and the potential role of PI3K/Akt signaling pathway in these effects (Fig. 1A,B and C). As shown in Fig. 2A and B, I/R caused significant myocardial injury. The infarct area (IA; white), the risk area (RA; red and white) and the left ventricular area (LV) are measured in each section. The IA was not observed in the sham group. Compared with the sham group (not shown), I/R induced significant increases in IA/RA, IA/LV, and RA/LV (*P* < 0.05). However, the infarct size of the H_2_ group was markedly reduced compared with that of the I/R group (*P* < 0.001). There were no significant differences in RA/LV, IA/RA, and IA/LV between the I/R and inhibitor groups (the I/R + HCH + LY, I/R + HCH + W, I/R + LY, and I/R + W groups) (*P* > 0.05), and the cardioprotective effects of H_2_ were completely abolished by PI3K inhibitors.

As shown in Supplementary Fig. 3, levels of serum myocardial enzymes increased significantly in the I/R group compared with those in the sham group (*P* < 0.05). However, after HCH therapy, the serum creatine kinase (CK), MB isoenzyme of CK (CK-MB), and cardiac troponin I (cTnI) levels were reduced significantly, but there were no significant differences in serum myocardial enzymes between the I/R and inhibitor groups (*P* > 0.05). Moreover, these parameters were significantly lower in the H_2_ group than in the hydrogen-rich saline group (*P* < 0.05).

**Figure 3:**
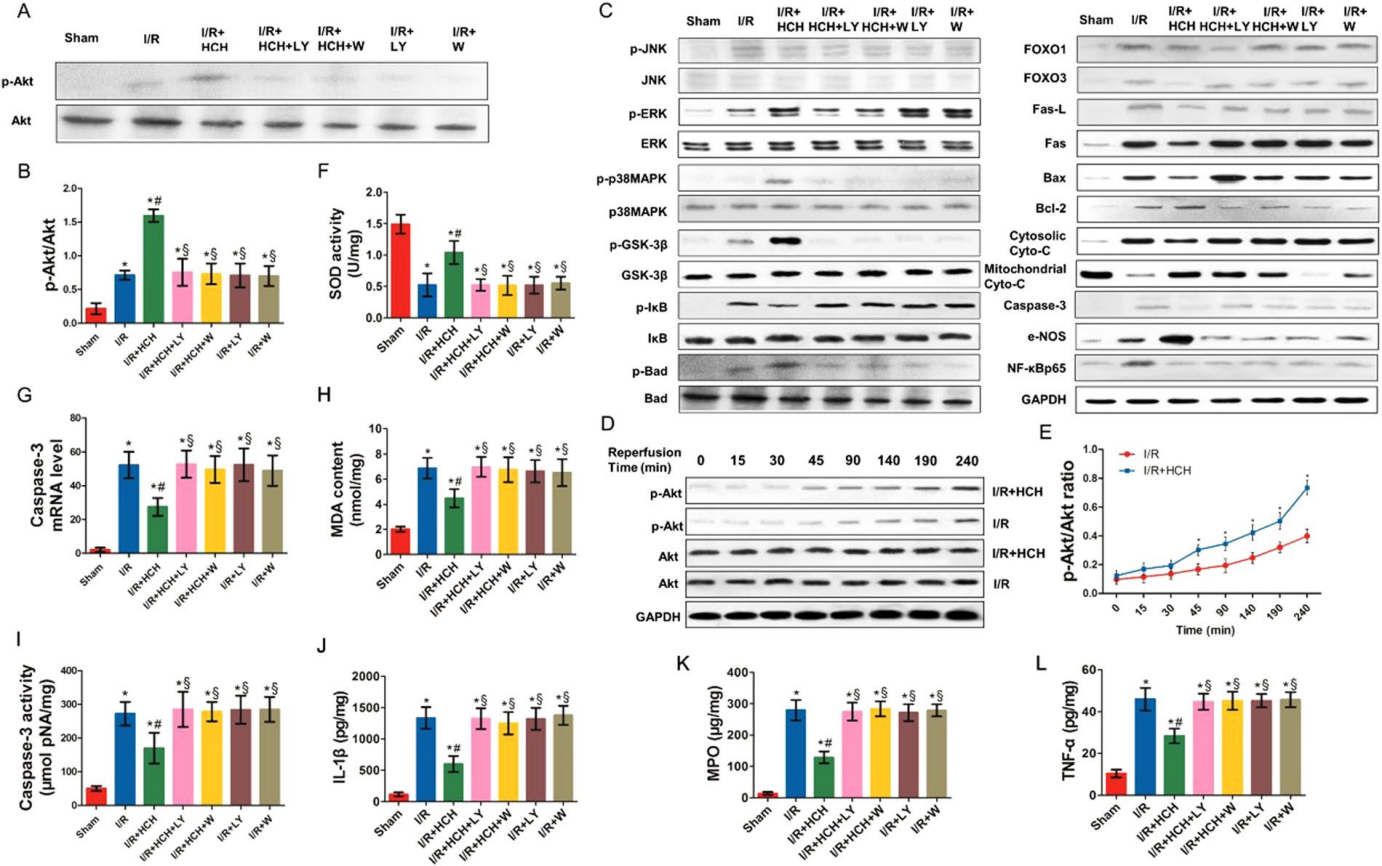
Protective effects of HCH following I/R in experiment 1. Mice were sacrificed after 4 h of reperfusion and the hearts were harvested for biochemical examination (n = 224). The expression of the following proteins was detected using western blotting: p-Akt, Akt, p-JNK, JNK, p-ERK, ERK, p-p38 MAPK, p38 MAPK, p-GSK-3β (Ser 9), GSK-3β, p-IκB, IκB, p-BAD, BAD, FOXO1, FOXO3, Fas-L, Fas, Bax, Bcl-2, cytoplasmic Cyto-c, mitochondrial Cyto-c, e-NOS, caspase-3, and NF-κBp65 (**A**,**C**). HCH significantly reduced the protein expression of p-JNK, p-IκB, FOXO1, FOXO3, Fas, Fas-L, Bax, cytoplasmic Cyto-c, caspase-3, and NF-κB, but markedly increased the protein expression of p-Akt, p-ERK, p-p38 MAPK, p-GSK-3β, p-BAD, Bcl-2, e-NOS, and mitochondrial cyt-c (**B**), Supplementary Fig. 4). HCH significantly reduced caspase-3 mRNA expression (**G**), MDA content (**H**), and caspase-3 activity (**I**), and markedly increased SOD activity (**H**). HCH significantly decreased the contents of TNF-α (**L**), IL-1β (**J**), and MPO (**K**) in the heart. PI3K inhibitor treatment markedly abolished the protective effects of HCH. The time course of Akt phosphorylation during ischemia and after reperfusion (**D**). Akt phosphorylation occurred earlier in HCH-treated animals (45 min after reperfusion) than in untreated I/R mice (90 min) (**E**). *P < 0.05 vs. sham; ^#^ *P* < 0.05 vs. I/R; ^§^ *P* < 0.05 vs. I/R + HCH.

The myocardial I/R mice presented with significantly reduced superoxide dismutase (SOD) activity, increased malondialdehyde (MDA) content, and an elevated number of cells positive for endothelial nitric oxide synthase (e-NOS) (Figs 3F,H, 2C,G) in the heart compared with mice in the sham group. In addition, the contents of tumor necrosis factor-α (TNF-α), interleukin-1β (IL-1β), and myeloperoxidase (MPO) increased dramatically in the heart after I/R (Fig. 3J,K,L). However, the contents of TNF-α, IL-1β, MPO, and MDA were significantly reduced and SOD activity increased markedly after HCH treatment (*P* < 0.01 or < 0.05 vs. the I/R group). The number of caspase-3- and NF-κB-positive cells significantly increased in the I/R group compared with that in the sham group, but decreased in the I/R + HCH group compared with that in the I/R group (*P* < 0.05) (Fig. 2C,F, and H). e-NOS-positive cells increased markedly in the I/R group (*P* < 0.05) and further increased after H_2_ treatment (*P* < 0.05 vs. the I/R group). Treatment with PI3K inhibitors completely abolished the anti-inflammatory effects of HCH.

To elucidate the mechanism underlying H_2_-induced protection against cardiomyocyte apoptosis, we tested the effects of HCH on the activation of JNK, extracellular regulated protein kinases (ERK), and p38 MAPK using western blotting. As shown in Fig. 3C and Supplementary Fig. 4, I/R injury increased JNK, ERK, and p38 MAPK phosphorylation (*P* < 0.05). However, HCH treatment decreased JNK phosphorylation and increased p38 MAPK and ERK phosphorylation after I/R (*P* < 0.05). Wortmannin (a potent PI3K inhibitor) and LY294002 (a strong PI3K inhibitor) abolished the effects of hydrogen on ERK, JNK, and p38 MAPK phosphorylation (*P* < 0.05). Mice treated with PI3K inhibitors showed similar levels of ERK, JNK, and p38 MAPK phosphorylation compared with mice in the I/R group (*P* > 0.05).

The heart rate differed among the groups 24 hours after surgery. The left ventricular systolic pressure (LVSP) increased markedly and the left ventricular end-diastolic pressure (LVEDP) decreased dramatically in the I/R + HCH group compared with those in the I/R group (*P* < 0.05). Moreover, the +dp/dt increased significantly and the −dp/dt decreased significantly after HCH treatment compared with those in the untreated I/R mice (*P* < 0.05). However, treatment with PI3K inhibitors blocked the cardioprotective effects of HCH. These findings suggest the improvement of cardiac function after HCH treatment (Supplementary Fig. 2).

### HCH affected Akt phosphorylation, modulated downstream effectors of Akt, and reduced I/R-induced apoptosis

As shown in Fig. 3A, total Akt expression in the heart remained unchanged after myocardial I/R and there was no difference in this regard between the I/R and I/R + HCH groups (*P* > 0.05). The level of p-Akt significantly increased after I/R injury (*P* < 0.05), but p-Akt levels increased after HCH treatment compared with the levels in I/R mice (*P* < 0.05). In addition, immunohistochemistry confirmed these findings. No p-Akt-positive cells were observed in the sham group, while the number of p-Akt-positive cells significantly increased in the I/R group. Compared with that in the I/R group, the number of p-Akt-positive cells in the I/R + HCH group increased significantly (*P* < 0.05). However, the effect of HCH on Akt phosphorylation was completely abolished by PI3K inhibitors. A time-dependent increase in Akt phosphorylation was also observed in the heart. Akt phosphorylation occurred earlier in I/R + HCH mice (45 min after reperfusion) than in untreated I/R mice (90 min) (*P* < 0.05) (Fig. 3D,E).

BAD and GSK-3β, two downstream effectors of Akt, are anti-apoptotic proteins, while Forkhead box O1 (FOXO1) and Forkhead box O3 (FOXO3) are apoptotic proteins. Moreover, e-NOS plays an important role in cardioprotection during I/R, and nuclear factor of kappa light polypeptide gene enhancer in B-cells inhibitor (IκB) is an anti-inflammatory protein. As shown in Fig. 3C and Supplementary Fig. 4, the levels of p-BAD, p-GSK-3β, e-NOS, and IκB were markedly increased after I/R compared with those of the sham group (*P* < 0.05). HCH treatment increased the levels of these proteins and affected the expression of their downstream effectors including Fas, Fas-L, Bax, Bcl-2, cytosolic Cytochrome c (cytosolic Cyto-c), mitochondrial Cytochrome c (mitochondrial Cyto-c), and NF-κB (*P* < 0.05). In addition, western blotting showed that the protein expression of FOXO1/3, factor associated suicide (Fas), and factor associated suicide ligand (Fas-L) increased significantly in the I/R group (*P* < *0.05* vs. sham), but decreased markedly in the I/R + HCH group (*P* < *0.05* vs. the I/R group). Treatment with PI3K inhibitors resulted in the effects of HCH treatment being abolished.

Apoptosis is the major mechanism of cell death following I/R injury. In our study, terminal deoxynucleotidyl transferase (TdT) dUTP nick-end labeling (TUNEL) staining and detection of caspase-3 mRNA, protein, and activity were employed to assess apoptosis. As shown in Figs 2C,D and Fig. 3C,G,I, I/R significantly increased cell apoptosis as evaluated by TUNEL staining as well as caspase-3 activity and mRNA levels compared with those in the sham group (*P* < 0.05). However, HCH treatment inhibited this increase in cell apoptosis (*P* < 0.05).

### The protective effects of HCH were attenuated by a selective Akt1 inhibitor

**Figure 4:**
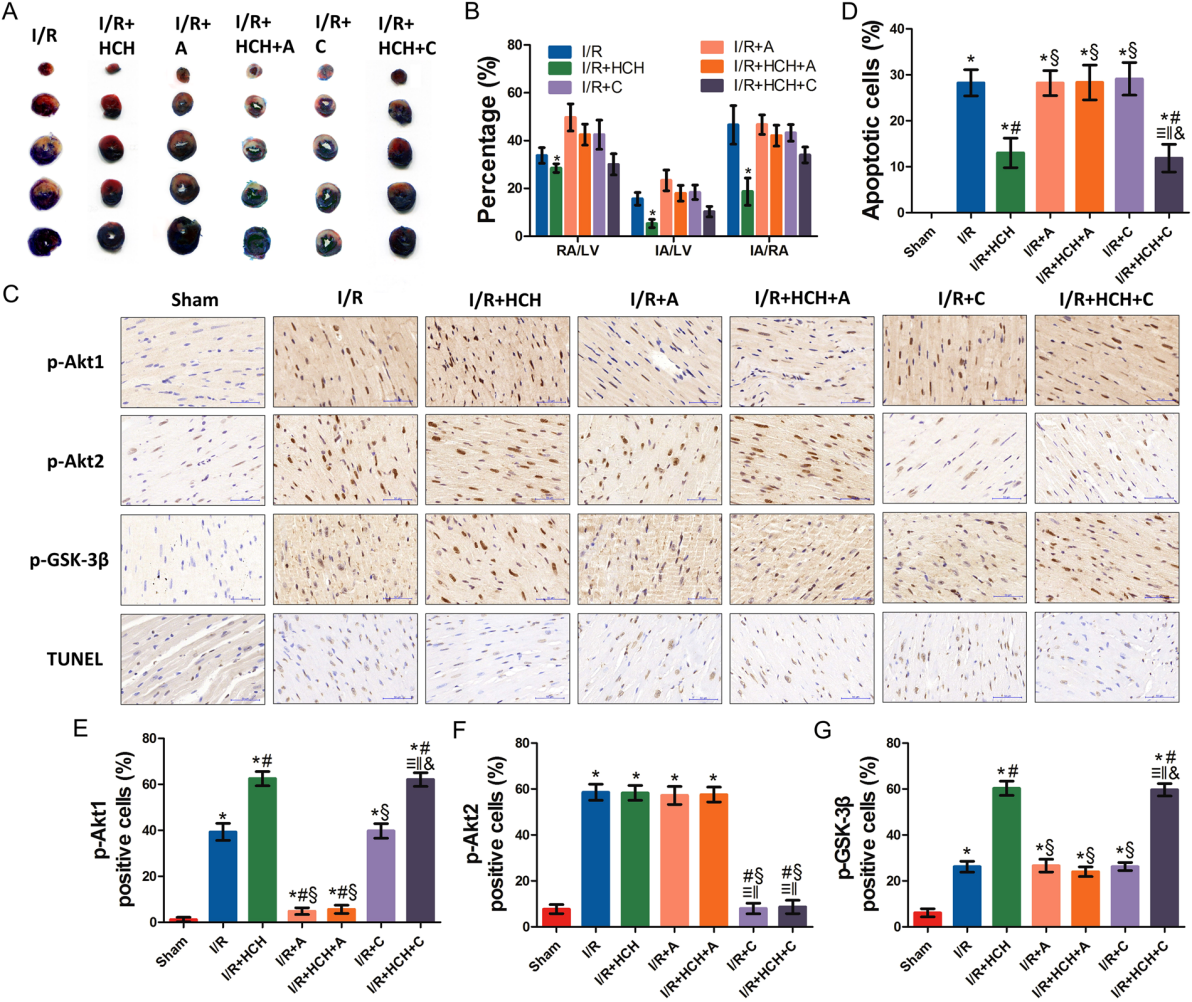
Morphological examination from experiment 2. Four hours after reperfusion, mice were sacrificed and the hearts were collected for determination of the infarct area (n = 56) and immunohistochemistry (n = 42). Treatment with an Akt1 inhibitor significantly increased the infarct area following I/R + HCH (*P* < 0.05). There were no significant differences in RA/LV, IA/RA, and IA/LV among the I/R, I/R + A, I/R + C, and I/R + HCH + A groups (*P* > 0.05) (**A**,**B**) and between the I/R + HCH and I/R + HCH + C groups (*P* > 0.05) (**A**,**B**). Immunohistochemistry was performed for p-Akt1, p-Akt2, and p-GSK-3β (**C**). Commercially available kits were used to perform TUNEL staining. Inhalation of HCH significantly increased the number of cells positive for p-Akt1 and p-GSK-3β and significantly decreased the number of cells positive for TUNEL (**D**,**E**,**G**). No significant differences were detected in the number of p-Akt2-positive cells among the I/R, I/R + HCH, I/R + A, and I/R + HCH + A groups (**F**). Treatment with Akt1 inhibitors markedly abolished the protective effects of HCH (**E**,**G**). *P < 0.05 vs. sham; ^#^ *P* < 0.05 vs. I/R; ^§^ *P* < 0.05 vs. I/R + HCH; ^≡^ *P* < 0.05 vs. sham; ^‖^ *P* < 0.05 vs. I/R; ^&^ *P* < 0.05 vs. I/R + HCH; magnification = ×400.

Studies have shown that Akt1 activation mediates the protective effects against myocardial I/R injury^27^. We evaluated the potential role of the Akt1 in the protective effects of hydrogen in the second part of animal experiment (Fig. 1D,E and F). As shown in Fig. 4A, when compared with the I/R + HCH group, the Akt1 selective inhibitor A-674563 induced significant increases in IA/RA, IA/LV, and RA/LV (*P* < *0.05*). However, RA/LV, IA/RA, and IA/LV remained unchanged upon CCT128930 treatment (*P* < *0.05*). In addition, there were no significant differences in this regard among the I/R, I/R + A, and I/R + C groups (*P* > 0.05).

As shown in Supplementary Fig. 3, serum levels of CK, CK-MB, and cTnI increased significantly in the I/R + HCH + A group compared with those in the I/R + HCH group (*P* < *0.05*). However, there were no significant differences between the I/R + HCH and I/R + HCH_ + _C groups (*P* > 0.05) and among the I/R, I/R + A, and I/R + C groups (*P* > 0.05).

**Figure 5:**
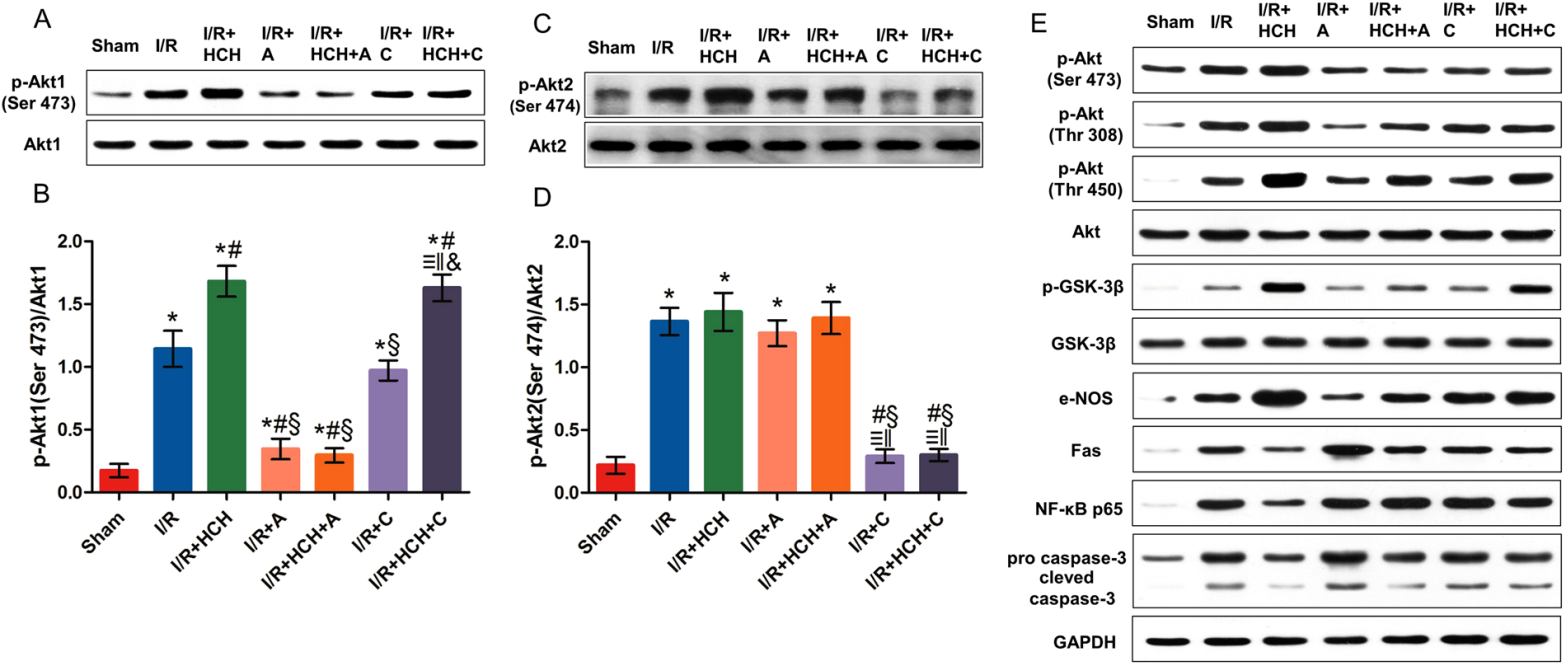
The roles of Akt1 and Akt2 in the protective effects of HCH in experiment 1. Mice were sacrificed after 4 h of reperfusion and the hearts were harvested for biochemical examinations (n = 56). The protein expression of p-Akt1 (Ser 473), Akt1, p-Akt2 (Ser 474), Akt2, p-Akt (Ser 473), p-Akt (Thr 308), p-Akt (Thr 450), Akt, p-GSK-3β, GSK-3β, e-NOS, Fas, NF-κB p65, pro-caspase-3, and cleaved caspase-3 was detected by western blotting (**A**,**C**,**E**). p-Akt1 expression increased markedly in the heart after 90-min HCH treatment. However, it was reduced significantly in the I/R + HCH + A group (**B**). There was no significant difference in p-Akt2 (Ser 474) among the I/R, I/R + HCH, I/R + HCH + A, and I/R + A groups, but p-Akt2 (Ser 474) levels were significantly decreased in the I/R + HCH + C and I/R + C groups (**D**). *P < 0.05 vs. sham; ^#^ *P* < 0.05 vs. I/R; ^§^ *P* < 0.05 vs. I/R + HCH; ^≡^ *P* < 0.05 vs. sham; ^‖^ *P* < 0.05 vs. I/R; ^&^ *P* < 0.05 vs. I/R + HCH.

As shown in Fig. 5, A-674563 markedly decreased p-Akt1 levels in the I/R + HCH + A group, and CCT128930 significantly decreased p-Akt2 levels in the I/R + HCH + C group. Western blotting revealed that there were significant differences in the protein expression of p-Akt1 (Ser 473), Akt1, p-Akt2 (Ser 474), Akt2, p-Akt (Ser 473), p-Akt (Thr 308), p-Akt (Thr 450), Akt, p-GSK-3β (Ser 9), GSK-3β, e-NOS, Fas, NF-κB p65, pro-caspase-3, and cleaved caspase-3 between the I/R + HCH and I/R + HCH + A groups. In addition, immunohistochemistry revealed that the number of p-Akt1-positive cells increased markedly in the heart after treatment with HCH for 90 min. However, the number of positive cells was reduced significantly in the I/R + HCH_ + _A group. There was no significant difference in the number of p-Akt2-positive cells among the I/R, I/R + HCH, I/R + HCH + A, and I/R + A groups, but it was decreased in the I/R + HCH + C and I/R + C groups.

Based on these *in vivo* experiments, it is likely that Akt1 mediates the protective effects of HCH on myocardial I/R injury. This was further investigated in the following *in vitro* experiments.

### 75% hydrogen exerted the greatest protective effect on neonatal mouse cardiomyocytes (NMCs) after Hypoxia/reoxygenation (H/R)

**Figure 6:**
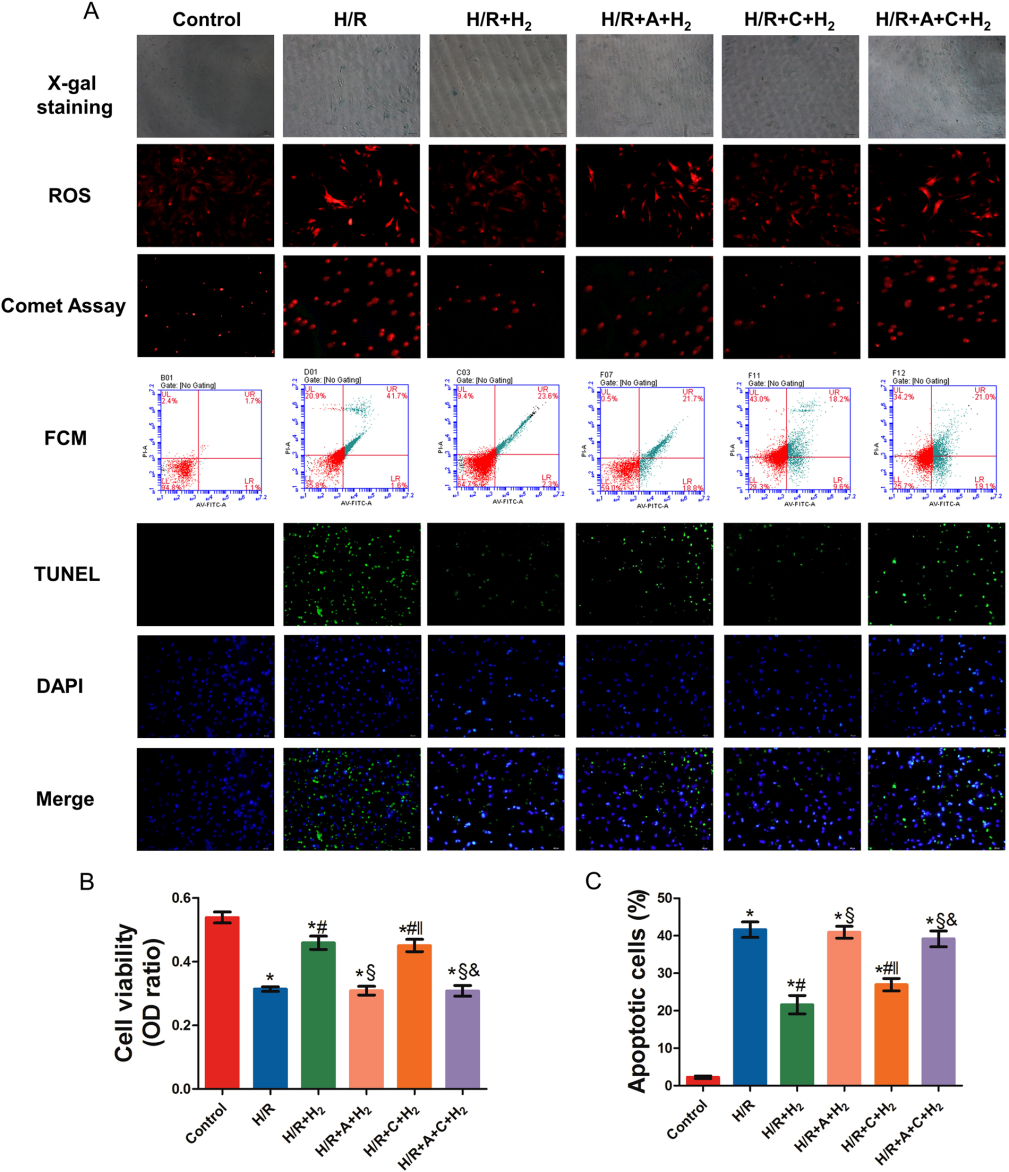
The protective effect of hydrogen gas at different concentrations *in vitro*. TUNEL staining, flow cytometry, and ROS detection were conducted to identify the optimal dose of H_2_ (**A**). The number of apoptotic cells decreased markedly after H_2_ treatment; significant differences were also noted among the different H_2_ groups and the largest reductions were observed in the H/R + 75% H_2_ group (*P* < 0.05). *P < 0.05 vs. control; ^#^ *P* < 0.05 vs. H/R; ^§^ *P* < 0.05 vs. H/R + 12.5% H_2_; ^‖^ *P* < 0.05 vs. H/R + 25% H_2_; ^&^ *P* < 0.05 vs. H/R + 50% H_2_.

TUNEL staining, flow cytometry, and reactive oxygen species (ROS) detection were conducted to identify the optimal dose of H_2_ at which its protective effects are maximized. During re-oxygenation, cells were independently exposed to 12%, 25%, 50%, and 75% H_2_. As shown in Fig. 6, TUNEL staining indicated that there were no apoptotic cells in the control group, and the number of apoptotic cells increased significantly in the H/R group. The number of apoptotic cells decreased markedly after H_2_ treatment, and a significant difference was also noted among the different H_2_ groups (*P* < 0.05). Flow cytometry after Annexin-V FITC/PI staining also revealed that H/R induced significant apoptosis compared with that in the control group, which was attenuated by H_2_ treatment in a dose-dependent manner. These results were consistent with those from TUNEL staining and indicate that H_2_ treatment may attenuate H/R-induced apoptosis in NMCs.

In addition, the ROS content increased significantly after H/R, while it decreased to different extents after H_2_ treatment. Moreover, the ROS content was significantly lower in the 75% H_2_ group than in the other H_2_ groups (*P* < 0.05). According to the above findings, 75% H_2_ conferred the best cardioprotective effects against H/R injury, so it was used in the following experiments.

### Akt1 phosphorylation mediated the protective effects of H_2_ on NMC H/R

**Figure 7:**
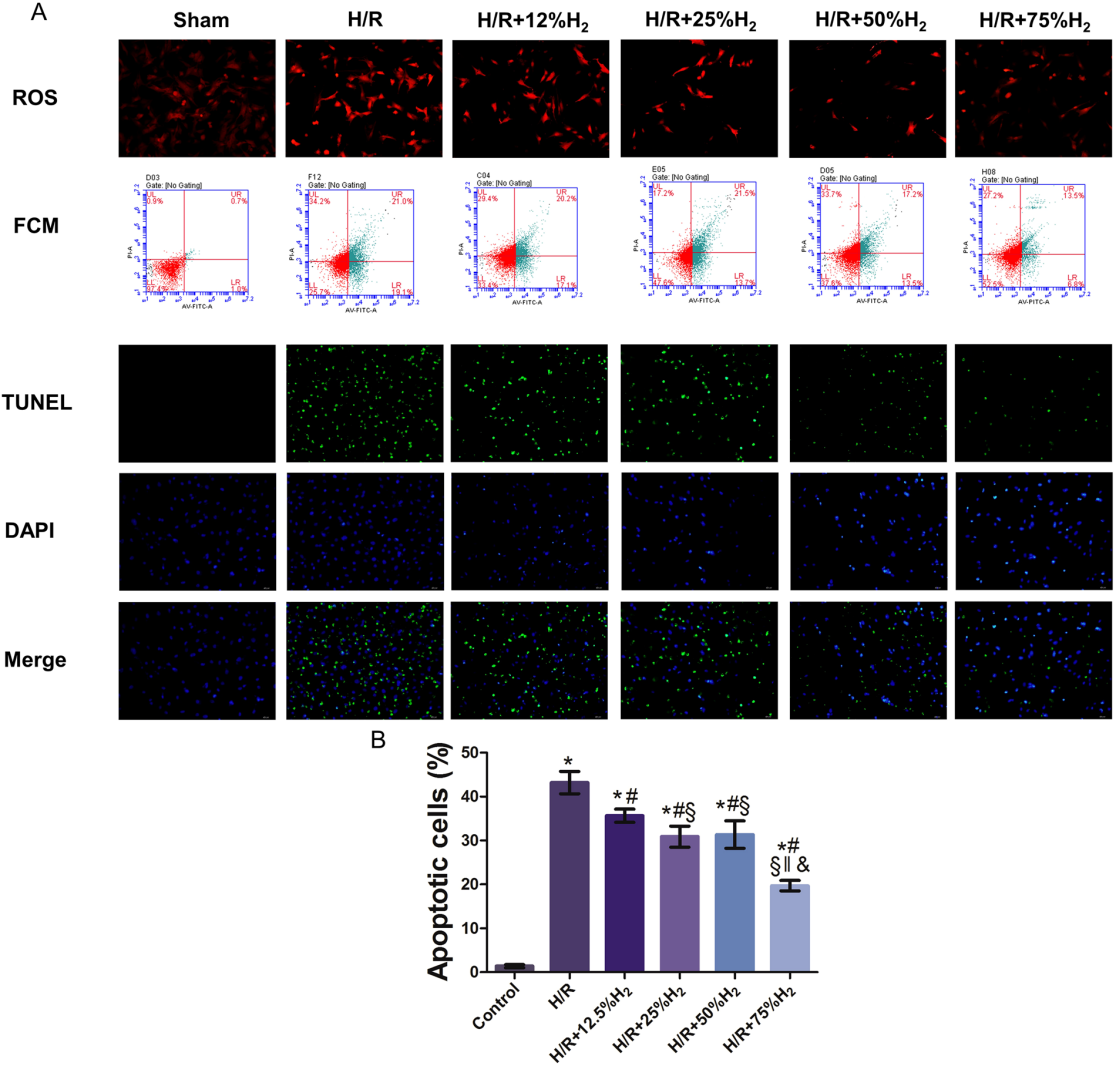
The roles of Akt1 and Akt2 in the protective effects of 75% H_2_. The MTT assay, X-gal staining, ROS detection, the comet assay, flow cytometry, and TUNEL staining were performed to evaluate the roles of Akt1 and Akt2 in the protective effects of 75% H_2_ in *in vitro* experiments. A-674563 significantly abolished the protective effects of H_2_. *P < 0.05 vs. control; ^#^ *P* < 0.05 vs. H/R; ^§^ *P* < 0.05 vs. H/R + HCH; ^‖^ *P* < 0.05 vs. H/R + HCH + A; ^&^ *P* < 0.05 vs. H/R + HCH + C.

As shown in Fig. 7, compared with the control group, the cell viability of the H/R, H/R + A + H_2_, and H/R + A + C + H_2_ groups was significantly reduced (*P* < 0.05). Exposure to 75% H_2_ significantly alleviated this effect (*P* < 0.001). In addition, there was no difference in cell viability between the H/R + H_2_ and H/R + C + H_2_ groups.

TUNEL staining, flow cytometry, and an alkaline comet assay were performed to assess cardiomyocyte apoptosis. The percentage of apoptotic cells in the H/R group was significantly increased compared with that in the control group. However, 75% H_2_ suppressed H/R-induced apoptosis of NMCs (*P* < 0.05). In comparison with the H/R + H_2_ group, pretreatment with A-674563 (H/R + A + H_2_ and H/R + A + C + H_2_) significantly increased the number of apoptotic cells.

**Figure 8:**
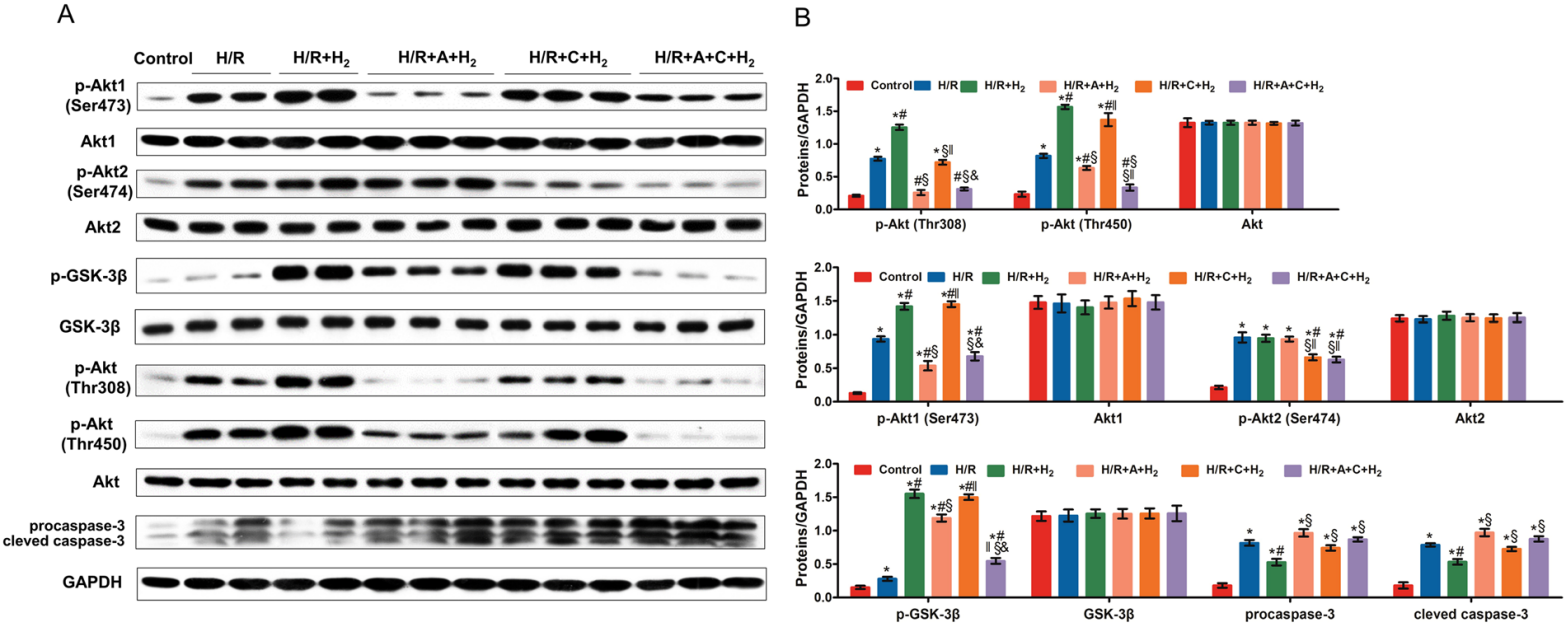
The roles of Akt1 and Akt2 in the protective effects of 75% H_2_. Western blotting was performed to determine the protein expression of p-Akt1 (Ser 473), Akt1, p-Akt2 (Ser 474), Akt2, p-Akt (Thr 308), p-Akt (Thr 450), Akt, p-GSK-3β, GSK-3β, pro-caspase-3, and cleaved caspase-3 (**A**,**B**). *P < 0.05 vs. control; ^#^ *P* < 0.05 vs. H/R; ^§^ *P* < 0.05 vs. H/R + HCH; ^‖^ *P* < 0.05 vs. H/R + HCH + A; ^&^ *P* < 0.05 vs. H/R + HCH + C.

As shown in Fig. 8, H_2_-treated NMCs presented with significantly reduced expression of pro-caspase-3 and cleaved caspase-3, and increased expression of p-Akt (Thr308 and Thr450), p-Akt1 (Ser473), and p-GSK-3β (Ser9). The expression levels of p-Akt, p-Akt1, and p-GSK-3β were reduced significantly, while the expression levels of pro-caspase-3 and cleaved caspase-3 were markedly increased in the presence of A-674563 pretreatment (*P* < 0.05). However, there was no significant difference in p-Akt2 expression among the H/R, H/R + H_2_, and H/R + A + H_2_ groups. ROS detection also indicated that A-674563 aggravated NMC oxidative damage after H/R.

## Discussion

It has been shown that hydrogen at a low concentration or hydrogen rich saline protects against myocardial I/R injury in rats^22–24,28^. However, the effects of HCH on myocardial I/R injury remain unclear and the mechanism underlying this protection should be explored. Our study showed for the first time that HCH after myocardial I/R injury ameliorated myocardial injury in a mouse I/R model (67% H_2_) and alleviated H/R-induced injury in NMCs *in vitro* (75% H_2_); these effects were attributed to activation of the PI3K/Akt1 pathway.

H_2_, the simplest element in nature, is a colorless and odorless diatomic gas. In 2007, studies by Ohsawa *et al*. demonstrated that H_2_ could selectively reduce peroxynitrite and hydroxyl radicals to alleviate cerebral I/R injury^22^. Since then, a number of studies on various animal models have demonstrated that H_2_ may exert protective effects on intestinal^29^, lung^30^, and myocardial I/R injuries^28^. In previous studies, H_2_ was administered via intraperitoneal injections of hydrogen-rich saline, drinking of hydrogen-rich water, or inhalation of H_2_ gas at low concentrations due to safety concerns. In recent years, a hydrogen/oxygen nebulizer (Asclepius, Shanghai, China) was developed to produce 66.7% H_2_ and 33.3% O_2_ by electrolyzing water. Studies from our group have also demonstrated that inhalation of a 66.7% H_2_ and 33.3% O_2_ mixture exerted protective effects on retinal I/R injury and glyoxylate-induced renal injury^25,31^. However, in both studies, the specific mechanism underlying the organ-protective effects of H_2_ at this high concentration was not investigated in depth.

In this study, we first confirmed that HCH could exert cardioprotective effects on I/R injury, as demonstrated by improvements in the infarct area, myocardial enzymes, and cardiac function, which was accompanied by the attenuation of inflammation and oxidative stress in the myocardium. In our pilot study, different duration of ischemia was induced in animals, and significant protective effects of hydrogen were observed after 4-h ischemia (short ischemia has limited infarct area and prolonged ischemia results in a high mortality). In this study, the cardioprotective effects were further confirmed.

Activation of the PI3K-Akt signaling pathway has been reported to play a critical role in myocardial protection during I/R via the regulation of cell growth, proliferation, survival, and endothelial cell migration as well as angiogenesis. Akt, a phosphorylation kinase that has more than 30 downstream substrates, has been shown to promote the survival of cardiomyocytes *in vitro* as well as to protect against I/R-induced injury in the mouse heart. Akt targets a wide variety of substrates via phosphorylation, including inactivation of the mitochondrial pro-apoptotic Bcl-2 family member BAD, FOXOs, and caspase-3; induction of e-NOS activity, which may reduce I/R injury through NO-induced inhibition of neutrophil infiltration; promotion of the nuclear translocation of NF-κB; and inhibition of GSK. In addition, phosphorylation by Akt has also been shown to down-regulate MAPK phosphatase-3 mRNA expression, resulting in prolonged phosphorylation of ERK and apoptosis signal-regulating kinase-1 (ASK1), which is believed to be the mediator of ROS-associated activation of JNK and p38-MAPK. Our previous study reported that treatment with hydrogen-rich saline protected the myocardium from I/R injury in a rat model, but the specific mechanism underlying this effect was not investigated further. We hypothesized that the PI3K/Akt pathway might mediate the cardioprotective effects of H_2_ in myocardial I/R injuries. Thus, the role of the PI3K/Akt pathway in the cardioprotective effects of HCH was investigated in this study. Our results showed that HCH increased Akt phosphorylation in cardiomyocytes after I/R injury, which was accompanied by activation of the downstream effectors of Akt, including JNK, ERK, p38 MAPK, BAD, GSK-3β, IκB, FOXO1/3, Fas-L, Fas, Bax, Bcl-2, cytosolic Cyto-c, mitochondrial Cyto-c, e-NOS, NF-κB, and caspase-3. In addition, pretreatment with pharmacological inhibitors of PI3K (LY294002 and wortmannin) abrogated the cardioprotective effects of HCH in mice with I/R injury, which was accompanied by the complete abolition of Akt phosphorylation.

The Akt subfamily comprises three mammalian isoforms (Akt1, Akt2, and Akt3)^9^. During mouse development, all three Akt isoforms are highly expressed in the heart; however, the expression of Akt3 in the heart was significantly lower than that of Akt1/2^27^. Thus, the activation of Akt1 and Akt2 was further investigated after HCH treatment *in vivo* and *in vitro*. Akt1 seems to be the most relevant in regulating cardiovascular functions, and it plays a critical role in the regulation of physiological cardiac growth, maladaptive pathological hypertrophy, and apoptosis^32^. In our study, HCH treatment did not change the expression of total Akt, but significantly increased p-Akt, suggesting the activation of Akt. In addition, levels of p-Akt1 markedly increased after HCH treatment, but there were no significant differences in p-Akt2 levels between the I/R and I/R + HCH (or H/R and H/R + HCH) groups. In addition, selective inhibition of Akt1 completely abolished the cardioprotective effects of HCH, but selective inhibition of Akt2 failed to affect the cardioprotective effects of HCH. These findings confirmed that Akt1, but not Akt2, is involved in cardioprotection by HCH.

Of note, how hydrogen acts on the PI3K is unclear. In recent years, other medical gases such as xenon and methane are speculated to be able to act on some proteins. For example, xenon can potently inhibit the N-methyl-D-aspartate (NMDA) receptors non-competitively^33^ and methane might confer an effect on membrane channels^34^. Thus, more studies are needed to elucidate the specific influence of hydrogen gas on these proteins.

However, there were still limitations in this study. First, we did not investigate the long-term cardioprotective effects of HCH or whether HCH treatment may prevent myocardial remodeling. Next, the pharmacological inhibitors used (PI3K inhibitors: LY294002 and wortmannin; Akt1 selective inhibitor: A-674563; Akt2 selective inhibitor: CCT128930) may not have completely inhibited phosphorylation, and our results should be further confirmed in animals with genetic modifications. In addition, whether other proteins related to cell survival are also involved in the protective effects and the extent of contribution of the PI3K/Akt pathway to these effects are still unclear. Lastly, we did not determine the *in vitro* H_2_ concentration of the medium equivalent to the H_2_ concentration of the HCH inhalation treatment used in mice and none of the antibodies use in this study was specifically proven for specificity.

In conclusion, to the best of our knowledge, we here showed for the first time that HCH can protect the myocardium against I/R injury *in vivo* and *in vitro* via activation of the PI3K/Akt signaling pathway. Moreover, Akt1, but not Akt2, mediates the PI3K-dependent cardioprotective effects of HCH. However, further investigation is warranted to determine whether HCH is applicable to the clinical treatment of myocardial I/R injury.

## Materials and Methods

### Mouse model of ischemia/reperfusion injury

Male C57BL/6 mice (20–25 g) were purchased from the Experimental Animal Center of the Second Military Medical University, Shanghai, China. The mice were subjected to myocardial I/R as described previously^35^. Briefly, the mice were anesthetized by intraperitoneal injections of pentobarbital (50 mg/kg) and ventilated using a small-animal ventilator (SAR-830; CWE, Ardmore, PA, USA). The left anterior descending (LAD) coronary artery was obstructed by ligation to induce ischemia for 45 min with 7-0 silk suture over a 1-mm polyethylene tube (PE-10). After ischemia, the coronary artery was reperfused by removing the suture. Mice were sacrificed after intraperitoneal anesthesia with pentobarbital (50 mg/kg) after 4-h reperfusion, and the hearts were harvested.

All protocols were approved by the Animal Care and Use Committee of the Second Military Medical University. All animal manipulations were performed in accordance with the recommendations of the Committee of the Care and Use of Laboratory Animals at the Second Military Medical University as well as the NIH guidelines.

### Administration of HCH

During reperfusion, the mice were exposed to HCH. In brief, they were placed in a closed plastic box, which was then flushed with 67% H_2_ and 33% O_2_ at a rate of 3,000 mL/min for 90 min. The AMS-H-01 hydrogen/oxygen nebulizer (Asclepius, Shanghai, China) was used, which was designed to electrolyze water to produce HCH. During the HCH exposure, the H_2_ concentration in the box was monitored with a hydrogen detector (MD2XP-3140; New Cosmos, Osaka, Japan). In groups without H_2_ treatment, the mice were exposed to 67% N_2_ and 33% O_2_.

### Determination of blood and myocardial hydrogen concentrations

Please see the supplementary materials.

### Animal experimental protocols

Experiment 1 was performed to determine the protective effects of HCH on myocardial I/R injury and to investigate the role of the PI3k-Akt pathway in the cardioprotective effects of HCH. Mice were randomly assigned to seven groups as follows: 1) sham (n = 56), mice underwent thoracotomy without LAD ligation; 2) I/R (n = 63), mice underwent myocardial I/R; 3) I/R + HCH (n = 59), mice underwent I/R and were then exposed to 67% H_2_ and 33% O_2_; 4) I/R + HCH + LY294002 (n = 61), mice were intravenously injected with the PI3k inhibitor LY294002 (40 mg/kg; Sigma-Aldrich, St. Louis, MO, USA) 1 h before myocardial ischemia and then exposed to 67% H_2_ and 33% O_2_; 5) I/R + HCH + wortmannin (n = 61), mice were intravenously injected with the PI3k inhibitor wortmannin (1 mg/kg; Selleck Chemicals, Houston, TX, USA) 1 h before myocardial ischemia and then exposed to 67% H_2_ and 33% O_2_; 6) I/R + LY294002 (n = 56), mice were intravenously injected with LY294002 (40 mg/kg, Sigma-Aldrich, St. Louis, MO, USA) 1 h before myocardial ischemia and then exposed to 67% N_2_ and 33% O_2_; and 7) I/R + wortmannin (n = 56), mice were intravenously injected with wortmannin (1 mg/kg; Selleck Chemicals) 1 h before myocardial ischemia and then exposed to 67% N_2_ and 33% O_2_. After 4 h of reperfusion, the hearts were harvested and stored at −80 °C for further analyses or fixed in 4% paraformaldehyde for immunohistochemistry and TUNEL staining.

Experiment 2 was undertaken to investigate the role of Akt1 in HCH-induced cardioprotection. Mice were randomized into seven groups as follows: 1) sham (n = 24); 2) I/R (n = 25); 3) I/R + HCH (n = 25); 4) I/R + A (n = 26), mice were intravenously injected with the Akt1 inhibitor A-674563 (100 mg/kg; Selleck Chemicals) 1 h before myocardial ischemia; 5) I/R + HCH + A (n = 25), mice were intravenously injected with A-674563 (100 mg/kg) 1 h before myocardial ischemia and then exposed to 67% H_2_ and 33% O_2_; 6) I/R + C (n = 26), mice were intravenously injected with the Akt2 inhibitor CCT128930 (50 mg/kg, Selleck Chemicals) 1 h before myocardial ischemia; and 7) I/R + HCH + C (n = 24), mice were intravenously injected with CCT128930 (50 mg/kg) 1 h before myocardial ischemia and then exposed to 67% H_2_ and 33% O_2_. After 4 h of reperfusion, the hearts were harvested and washed with ice-cold normal saline. Then, the hearts were cut into 2-mm cross sections and fixed in 4% paraformaldehyde for immunohistochemistry, immunofluorescent staining, and TUNEL staining.

LY294002, wortmannin, A-674563, and CCT128930 were independently dissolved in dimethyl sulfoxide (DMSO). In the control group, the mice were injected with the same volume of DMSO.

### Isolation of cardiomyocytes from neonatal mice

Primary cardiomyocytes were prepared from neonatal C57BL/6 mice (1–3 days old) as described previously^36^. Briefly, after intraperitoneal anesthesia with pentobarbital (50 mg/kg), the hearts were collected, and the ventricles of newborn mice were isolated aseptically, digested in 0.3% trypsin and collagenase, and purified. The cells were maintained in Dulbecco’s Modified Eagle Medium (DMEM) F-12 (Life Technologies, Grand Island, NY, USA), 20% gold fetal bovine serum, and 1% penicillin/streptomycin (Life Technologies). The cells were grown in an environment with 95% air and 5% CO_2_ at 37 °C.

### *In vitro* experiments

Hypoxia/reoxygenation was performed in cells two days after separation as described previously^37^. For H/R, cells were grown in Tyrode’s buffer (130 mM NaCl, 5 mM KCl, 10 mM HEPES, 1 mM MgCl_2_, 1 mM CaCl_2_, pH 7.4) in an incubator (BioSpherix) with 5% CO_2_ and 95% N_2_ for 3 h (hypoxia), and then exposed to an environment with 20% O_2_, 5% CO_2,_ and 75% N_2_ for 4 h (reoxygenation). To explore the optimal dose of H_2_, cardiomyocytes were randomly divided into the following six groups: 1) control group, cells were maintained in an environment with 20% O_2_, 5% CO_2_, and 75% N_2_ at 37 °C; 2) H/R group, cells were maintained in a hypoxic environment for 3 h and subsequently received reoxygenation for 4 h; 3) H/R + 12.5% H_2_ group, cells received 3 h of hypoxia and were then exposed to 12.5% H_2_ (20% O_2_, 5% CO_2_, 12.5% H_2_, and 62.5% N_2_) for 4 h; 4) H/R + 25% H_2_ group, cells received 3 h of hypoxia and were then exposed to 25% H_2_ (20% O_2_, 5% CO_2_, 25% H_2_, and 50% N_2_) for 4 h; 5) H/R + 50% H_2_ group, cells received 3 h of hypoxia and were then exposed to 50% H_2_ (20% O_2_, 5% CO_2_, 50% H_2_, and 25% N_2_) for 4 h; and 6) H/R + 75% H_2_ group, cells received 3 h of hypoxia and were then exposed to 75% H_2_ (20% O_2_, 5% CO_2_, and 75% H_2_) for 4 h. Flow cytometry, TUNEL staining, and ROS measurements were performed to confirm the optimal dose of H_2_. After that, the role of Akt1 and Akt2 in the protective effects of H_2_ was further investigated. Cardiomyocytes were randomly assigned to six groups: 1) control group, cells were maintained in an environment with 20% O_2_, 5% CO_2_, and 75% N_2_ at 37 °C; 2) H/R group, cells received 3 h of hypoxia and 4 h of reoxygenation; 3) H_2_ group (H/R + H_2_), cells received 3 h of hypoxia and 4 h of exposure to 75% H_2_; 4) Akt1 inhibition group (H/R + A + H_2_), cells were treated with 5 μM A-674563 followed by H/R and H_2_ treatment; 5) Akt2 inhibition group (H/R + C + H_2_), cells were treated with 5 μM CCT128930 followed by H/R and H_2_ treatment; and 6) Akt1 and Akt2 inhibition group (H/R + A + C + H_2_), cells were treated with 5 μM A-674563 and 5 μM CCT128930 followed by H/R and H_2_ treatment.

### Triphenyltetrazolium chloride staining

Following 4 h of reperfusion, the mice were injected with 0.3 mL of 2% Evan’s Blue dye. The hearts were subsequently harvested, frozen, sliced, and incubated with 1% tetrazolium chloride at 37 °C for 20 min in the dark. After being fixed in 10% paraformaldehyde, the infarct area was measured using the Image-Pro Plus 6.0 software (Media Cybernetics, Silver Spring, MD, USA). Each section was scanned using an HP Scanjet 4890 (HP, Palo Alto, CA, USA), and IA and RA were measured. The ratios of the RA to the left ventricular area (RA/LV) and of the IA to the RA (IA/RA) were independently calculated and expressed as percentages.

### Detection of myocardial enzymes

Four hours after surgery, the blood of the mice from experiment 1 and of the I/R + 5 mL/kg hydrogen-rich saline group was collected and centrifuged at 2500 × g for 5 min at 4 °C, followed by serum collection. The production of hydrogen-rich saline was performed as described previously^23^. The levels of myocardial enzymes CK and CK-MB were measured using the colorimetric method, in accordance with the manufacturer’s instructions (Nanjing Jiancheng Bioengineering Institute, Nanjing, China). Serum cTnI was determined using an immunoassay (Roche Diagnostics Elecsys 2010, Mannheim, Germany).

### Extraction of the mitochondrial fraction

The hearts were collected and homogenized immediately in lysis buffer after reperfusion, and the cytosolic and mitochondrial fractions were separated using a Tissue Mitochondria Isolation kit (Beyotime Institute of Biotechnology, Jiangsu, China). The final precipitate was re-suspended in Mitochondrial Extraction Buffer Mix and analyzed as the mitochondrial fraction.

### Determination of oxidative parameters

In experiment 1, the hearts were harvested, washed, homogenized, and centrifuged at 3000 × g for 20 min at 4 °C. After quantification of the protein concentration using the bicinchoninic acid (BCA) method, SOD activity (U/m) and MDA concentration (nmol/mg protein) were determined using commercially available kits (Nanjing Jiancheng Bioengineering Institute, Nanjing, China).

### Detection of caspase-3 activity

The caspase-3 activity of the cytosolic fraction was measured using a caspase-3 activity colorimetric assay kit, in accordance with the manufacturer’s instructions (Beyotime Institute of Biotechnology, Jiangsu, China). Briefly, 100 μg of cell lysate was added to a 96-well plate, followed by incubation with 20 μg of caspase-3 substrate (Ac-DEVD-pNA) for 2 h at 37 °C. After incubation in the dark for 4 h, the absorbance was measured at 405 nm using an automatic spectrophotometer (ELx800; BioTek Instruments, Winooski, VT, USA). Caspase-3 activity was calculated using a standard curve (μmol pNA/mg protein).

### Measurement of tumor necrosis factor-α, interleukin-1β, interleukin-6, and myeloperoxidase contents

Heart supernatants were collected and used to measure the contents of TNF-α, IL-1β, and MPO using commercial enzyme-linked immunosorbent assay (ELISA) kits (R&D Systems, Minneapolis, MN, USA), following the manufacturer’s instructions. The absorbance was measured at 450 nm using a microplate reader (ELx800; BioTek Instruments, Winooski, VT, USA) and the contents of these proteins were calculated using standard curves.

### Quantitative RT-PCR

Total RNA was extracted from the heart using TRIzol reagent (Invitrogen, Carlsbad, CA, USA) following the manufacturer’s instructions, and processed for quantitative reverse transcriptase polymerase chain reaction (RT-PCR). The primers used for PCR were as follows: *caspase-*3, 5′-GCG GTA TTG AGA CAG ACA GTG GAA C-3′ (forward) and 5′-GCG GTA GAG TAA GCA TAC AGG AAG T-3′ (reverse; product size, 91 bp); and *β-actin* 5′-CAC TAT CGG CAA TGA GCG GTT CC-3′ (forward) and 5′-CAG CAC TGT GTT GGC ATA GAG GT-3′ (reverse; product size, 154 bp). Total RNA was reverse-transcribed into cDNA in a 50-μL mixture containing 2 μg of total RNA at 95 °C for 10 min, followed by 30 cycles at 94 °C for 30 s, 58 °C for 30 s, and 72 °C for 30 s. Real-time PCR was performed in triplicate using a real-time PCR system (StepOne Plus^TM^, Foster City, CA, USA), and the mRNA expression of target genes was normalized to that of β-actin.

### Western blotting

The levels of the following proteins from the left ventricle were measured by western blotting with antibodies from Cell Signaling Technology (Danvers, MA, USA): phosphorylated (p)-Akt, Akt, p-JNK, JNK, p-ERK, ERK, p-p38MAPK, p38MAPK, p-GSK-3β, GSK-3β, p-IκB, IκB, p-BAD, BAD, FOXO1, FOXO3, Fas, Fas-L, Bax, Bcl-2, mitochondrial and cytosolic cytochrome c, caspase-3, e-NOS, and NF-κB for experiment 1; and p-Akt1 [Ser 473], p-Akt1, p-Akt2 [Ser 474], p-Akt2, p-Akt [Ser 473], p-Akt [Thr 308], p-Akt [Thr 450], Akt, e-NOS, Fas, NF-κB, and pro and cleaved caspase-3 for experiment 2. The total protein in each sample was quantified and subsequently separated using sodium dodecyl sulfate-polyacrylamide gel electrophoresis (SDS-PAGE). The proteins were transferred onto polyvinylidene difluoride (PVDF) membranes (Bio-Rad Laboratories, Hercules, CA, USA), which were blocked and incubated with specific primary antibodies (1:1000) overnight at 4 °C. Then, the membranes were washed and incubated with horseradish peroxidase (HRP)-conjugated secondary antibodies (1:5000) in 5% non-fat milk in Tris-buffered saline with Tween 20 (TBS-T) at room temperature for 45 min. Bands were visualized with enhanced chemiluminescence (ECL) detection reagents (Bestbio, Shanghai, China) using an ECL assay, in accordance with the manufacturer’s instructions. The resulting bands were quantified using the Quantity One Analysis Software (Bio-Rad). The protein expression was normalized to that of GAPDH as the relative expression of a specific protein.

### Histological examination

The hearts were harvested and processed for immunohistochemistry and immunofluorescence staining of p-Akt, caspase-3, NF-κB, e-NOS, p-Akt1, p-Akt2, and p-GSK-3β. Briefly, the hearts were fixed in 4% paraformaldehyde, embedded in paraffin, cut into 4-μm sections, and then processed for immunohistochemistry as previously described^38^. Under a microscope, images were captured at ×400 magnification and five fields were randomly selected from the left ventricular area; positive cells contained brown granules. For quantification, the mean optical density (OD) was calculated and analyzed using the Image-Pro Plus 6.0 software (Media Cybernetics). The percentage of positive cells was calculated as follows: (number of positive cells in a field)/(number of total cells in a field) ×100.

### TUNEL staining

In an *in vitro* experiment, after 4 h of reoxygenation, apoptotic cells were quantified following TUNEL staining with a fluorescence detection kit (Roche Molecular Biochemicals, Indianapolis, IN, USA). Following standard protocols, TUNEL staining was performed in accordance with the manufacturer’s instructions.

Apoptosis of NMCs was assessed using the TUNEL detection kit, in accordance with the manufacturer’s instructions. Cells were fixed with 4% formaldehyde in PBS for 25 min at 4 °C and incubated with Roche’s reagent.

Fluorescent cells were observed with a fluorescence microscope (BX-51; Olympus, Tokyo, Japan) equipped with a digital camera, and images were taken at a magnification of ×400. The number of apoptotic cells and the number of total cells were determined using the Image-Pro Plus 6.0 software (Media Cybernetics). Ten randomly selected fields per section were analyzed and the apoptosis index was calculated.

### Hemodynamic measurements

Please see the supplementary materials.

### Cell viability

Cell viability was determined using a methyl thiazolyl tetrazolium (MTT) assay, in accordance with the manufacturer’s instructions. Cells were seeded into 96-well plates at 1 × 10^4^ cells/well, and the MTT solution was added into each well (5 mg/mL), followed by incubation for 4 h at 37 °C. Cell viability was measured by detecting the absorbance at 570 nm.

### Measurement of intracellular ROS

Intracellular ROS was measured using dihydroethidium (DHE, Sigma). Briefly, cells were seeded into 96-plates at 5 × 10^5^ cells/well. After incubation with 5 μM DHE at 37 °C for 20 min in the dark, cells were washed for 5 min in PBS three times to remove the unbound DHE. Fluorescence was detected with a microscope (BX-51; Olympus, Tokyo, Japan) equipped with a digital camera.

### X-gal staining

X-gal (5-bromo-4-chloro-3-indolyl-β-D-galactopyranoside) staining was employed to detect the active β-galactosidase enzyme, indicative of senescent cells. After 7 h of experimental processing, cells were digested in 0.25% trypsin and seeded into 6-well plates at 5 × 10^5^ cells/well, followed by incubation at 37 °C for 24 h. Subsequently, cells were washed three times with PBS and stained with a staining solution containing 400 μg/mL X-gal, 4 mM MgCl_2_, 4 mM potassium ferrocyanide, and 4 mM potassium ferricyanide in PBS for 50 min at 37 °C, in accordance with the manufacturer’s instructions. The stained colonies were then visualized and automatically counted under an Olympus microscope using the Image-Pro Plus software (Media Cybernetics).

### Flow cytometry

Apoptotic cells were detected after Annexin V-FITC/PI double staining using flow cytometry. Briefly, cells were digested in 0.25% trypsin-EDTA and single cell suspensions were prepared. After washing twice with PBS, Annexin V-FITC and PI were added to cells, followed by incubation in the dark for 15 min at 25 °C. Flow cytometry was performed using a BD FACSCalibur^TM^ flow cytometer (BD Biosciences, San Jose, CA, USA) within 1 h, and data were analyzed using the Cell Quest software (BD Biosciences). Staining indicated the following: Annexin V+/PI−, early apoptotic cells; Annexin V+/PI+, late apoptotic cells; and Annexin V−/PI− normal cells. Early- and late-stage apoptotic cells were counted, and the proportion of apoptotic cells was calculated.

### Alkaline comet assay

An alkaline comet assay was used for the detection of DNA fragmentation associated with apoptosis. DNA damage was determined using a commercially available kit for single cell electrophoresis using the detailed protocol described previously^39^.

### Statistical analysis

Quantitative data are expressed as the mean ± standard deviation (SD) for normally distributed variables and as median (quartiles) for non-normally distributed variables. Comparisons of quantitative data were performed using one-way analysis of variance (ANOVA) followed by the Student-Newman-Keuls (SNK) test. All statistical analyses were performed using the SPSS software version 21.0 (SPSS, Chicago, IL, USA). A value of *P* < 0.05 was considered to indicate statistical significance.

## Electronic supplementary material

supplementary materials
