# Supplementary material for: High-concentration hydrogen protects mouse heart against ischemia/reperfusion injury through activation of thePI3K/Akt1 pathway
Source: Sci Rep. 2017 Nov 1;7:14871. doi: 10.1038/s41598-017-14072-x (PMC5665927; doi:10.1038/s41598-017-14072-x)
Supplement: Supplementary file 1 — supplementary materials [file 41598_2017_14072_MOESM1_ESM.pdf]

High-concentration hydrogen protects mouse heart against ischemia/reperfusion injury through activation of the PI3K/Akt1 pathway

Ouyang Chen, [1, 2, a] Zhiyong Cao, [5 a] He Li, [1, 2] Zhouheng Ye, [1] Rongjia Zhang, [1] Ning Zhang, [1] Junlong Huang, [1] Ting Zhang [1], Liping Wang, [6] Ling Han, [4] Wenwu Liu, [3,*] and Xuejun Sun.[1,*]

Animal experimental protocols

Experiment 1 was to determine the protective effects of HCH on myocardial I/R injury and investigate the role of PI3k-Akt pathway in the carioprotection of HCH. Mice were randomly assigned into 7 groups: (a) Sham (n=56): Mice underwent thoracotomy without LAD ligation; (b) I/R (n=63): Mice underwent myocardial I/R; (c) HCH (n=59): Mice underwent I/R and were then exposed to 67% H_2 and 33% O_2; (d) I/R + H_2 + LY294002 (n=61): Mice were intravenously injected with PI3k inhibitor LY294002 (40 mg/kg, Sigma-Aldrich, St Louis, MO, USA) 1 h before myocardial ischemia and were then exposed to 67% H_2 and 33% O_2; (e) I/R + H_2 + Wortmannin (n=61) : Mice were intravenously injected with PI3k inhibitor Wortmannin (1 mg/kg, Selleckchem, Houston, Texas, USA) 1 h before myocardial ischemia and were then exposed to 67% H_2 and 33% O_2; (f) I/R + LY294002 (n=56): Mice were intravenously injected with LY294002 (40 mg/kg, Sigma-Aldrich, St Louis, MO, USA) 1 h before myocardial ischemia and were then exposed to 67% N_2 and 33% O_2; (g) I/R + Wortmannin (n=56): Mice were intravenously injected with Wortmannin (1 mg/kg, Selleckchem, Houston, TX, USA) 1 h before myocardial ischemia and were then exposed to 67% N_2 and 33% O_2. After 4-h reperfusion, the hearts were harvested and stored at −80°C for further analyses or fixed in 4% buffered paraformaldehyde for immumohistochemistry and TUNEL staining.

Experiment 2 was to investigate the role of Akt1 phosphorylation in HCH-induced cardioprotection. Mice were randomized into seven groups: (a) Sham (n=24); (b) I/R (n=25); (c) HCH (n=25); (d) I/R + A-674563 (n=26): Mice were injected with Akt1 inhibitor A-674563 (100 mg/kg, Selleckchem, Houston, TX, USA) 1 h before myocardial ischemia; (e) I/R + HCH + A-674563 (n=25): Mice were injected with A-674563 (100 mg/kg) 1 h before myocardial ischemia and were then exposed to 67% H_2 and 33% O_2; (f) I/R + CCT128930 (n=26): Mice were injected with Akt2 inhibitor CCT128930 (50 mg/kg, Selleckchem, Houston, TX, USA) 1 h before myocardial ischemia; (g) I/R + H_2 + CCT128930 (n=24): Mice were injected with CCT128930 (50 mg/kg) 1 h before myocardial ischemia and were then exposed to 67% H_2 and 33% O_2. After 4-h reperfusion, the hearts were harvested and washed with ice-cold normal saline. Then, the hearts were cut into 2 mm cross-sections and then fixed in 4% buffered paraformaldehyde for further immumohistochemistry, immunofluorescent staining and TUNEL staining.

LY294002, Wortmannin, A-674563 and CCT128930 were independently dissolved in dimethyl sulfoxide (DMSO). In control group, mice were injected with DMSO of the same volume.

Determination of blood and myocardial hydrogen concentrations

The concentration of H_2 in blood was determined in normal mice. Blood was collected from the femoral artery and vein soon after 90 min inhalation of 66.7% H_2. Each blood sample was placed in a tube filled with nitrogen, and 1 ml of gas above the blood was collected for the measurement of H_2 concentration by gas chromatography (Gas Chromatography-9860, Qiyang, Shanghai, China). The H_2 concentration was calculated as follow: blood H_2 concentration (μl/L) = A × B/C; where A is the measurement value of H_2, B is the total volume of the gas above the blood (19 ml) and C is the volume of blood collected.

To measure the H_2 concentration of the heart tissue, mice were divided into three groups: sham group (sham, n=6), I/R group (I/R + 67% N_2 and 33% O_2, n=6) and I/R + H_2 group (I/R + 67% O_2 and 33% H_2, n=6). The hearts were harvest soon after 90 min inhalation of 66.7% H_2 and immediately placed into gentleMACS tubes (Miltenyi Biotec, Bergisch Gladbach, Germany) filled with nitrogen and homogenized using the gentleMACS™ Dissociator (Miltenyi Biotec, Bergisch Gladbach, Germany). After that, 1 ml of gas in the tube was collected for the measurement of H_2 concentration by gas chromatography (Gas Chromatography-9860, Qiyang, Shanghai, China). The H_2 concentration was calculated as follows: myocardial H_2 concentration (μl/kg) = A×D/E; where A is the measurement value of H_2, D is the gas volume of the gentleMACS tube (24 ml) and D is the weight of myocardium.

Hemodynamic measurements

Twenty-four hours after surgery, a high fidelity micro manometer catheter (Millar Instruments, Houston, USA) connected to a pressure sensor was inserted into the right carotid artery to record the arterial blood pressure and heart rate. The catheter was advanced into the lumen of left ventricular for recording the left ventricular pressures (LVP). LVSP, LVDP, heart rate (HR) and the maximal rate of the rise in LV pressure (±dP/dtmax) were calculated from the continuously recorded LVP signals.

***in vitro* experiment**

Hypoxia/reoxygenation (H/R) was done in cells at 2 days after separation as described previously [29]. For H/R, cells were grown in Tyrode buffer (NaCl 130 mM, KCl 5 mM, HEPES 10 mM, $MgCl_2$ 1 mM, $CaCl_2$ 1 mM, pH 7.4) in an incubator (BioSpherix) with 5% CO_2 and 95% N_2 for 3 h (hypoxia), and then exposed to an environment with 20% O_2, 5% CO_2 and 75% N_2 for 4 h (reoxygenation). To explore the optimal dose of H_2, cardiomyocytes were randomly divided into the following group: (a) Control group: cells were maintained in an environment with 20% O_2, 5% CO_2 and 75% N_2 at 37 ℃; (b) H/R

group: cells were maintained in a hypoxic environment for 3 h and received reoxygenation for 4 h; (c) 12.5% H_2 group: cells received 3-h hypoxia and then exposed to 12.5% H_2 (20% O_2, 5% CO_2, 12.5% H_2, 62.5% N_2) for 4 h; (d) 25% H_2 group: cells received 3-h hypoxia and then exposed to 25% H_2 (20% O_2, 5% CO_2, 25% H_2, 50% N_2) for 4 h; (e) 50% H_2 group: cells received 3-h hypoxia and then exposed to 50% H_2 (20% O_2, 5% CO_2, 50% H_2, 25% N_2) for 4 h; (f) 75% H_2 group: cells received 3-h hypoxia and then exposed to 75% H_2 (20% O_2, 5% CO_2, 75% H_2) for 4 h. Flow cytometry, TUNEL staining and ROS measurement were performed to confirm the optimal dose of H_2. After that, the role of Akt1 and Akt2 in the protective effects of H_2 was further investigated. Cardiomyocytes were assigned randomly into 6 groups: (a) Control group: cells were maintained in an environment with 20% O_2, 5% CO_2 and 75% N_2 at 37℃; (b) H/R group: cells received 3-h hypoxia and 4-h reoxygenation; (c) H_2 group (H/R + H_2): cells received 3-h hypoxia and 4-h exposure to 75% H_2; (d) Akt1 inhibition group (H/R + A-674563 + H_2): cells were treated with 5 μM A-674563, followed by H/R and H_2 treatment; (e) Akt2 inhibition group (H/R + CCT128930 + H_2): cells were treated with 5 μM CCT128930, followed by H/R and H_2 treatment; (f) Akt1 and Akt2 inhibition group (H/R+ A-674563 + CCT128930 + H_2): cells were treated with 5 μM A-674563 and 5 μM CCT128930, followed by H/R and H_2 treatment.

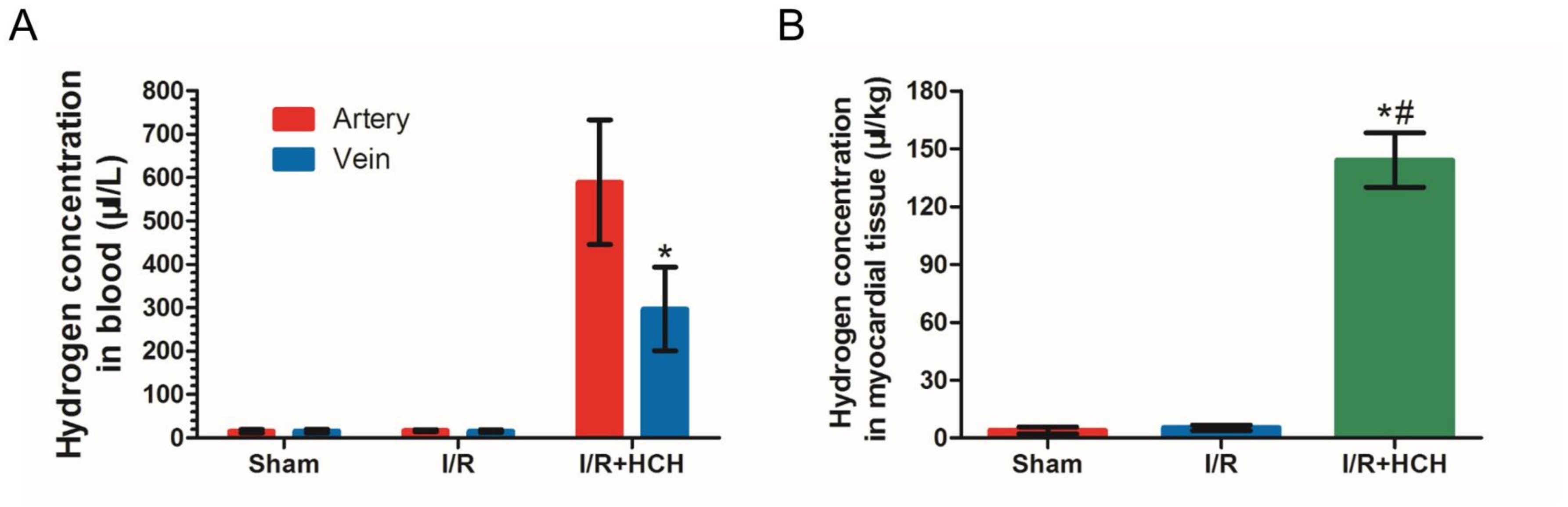

Supplementary Figure 1

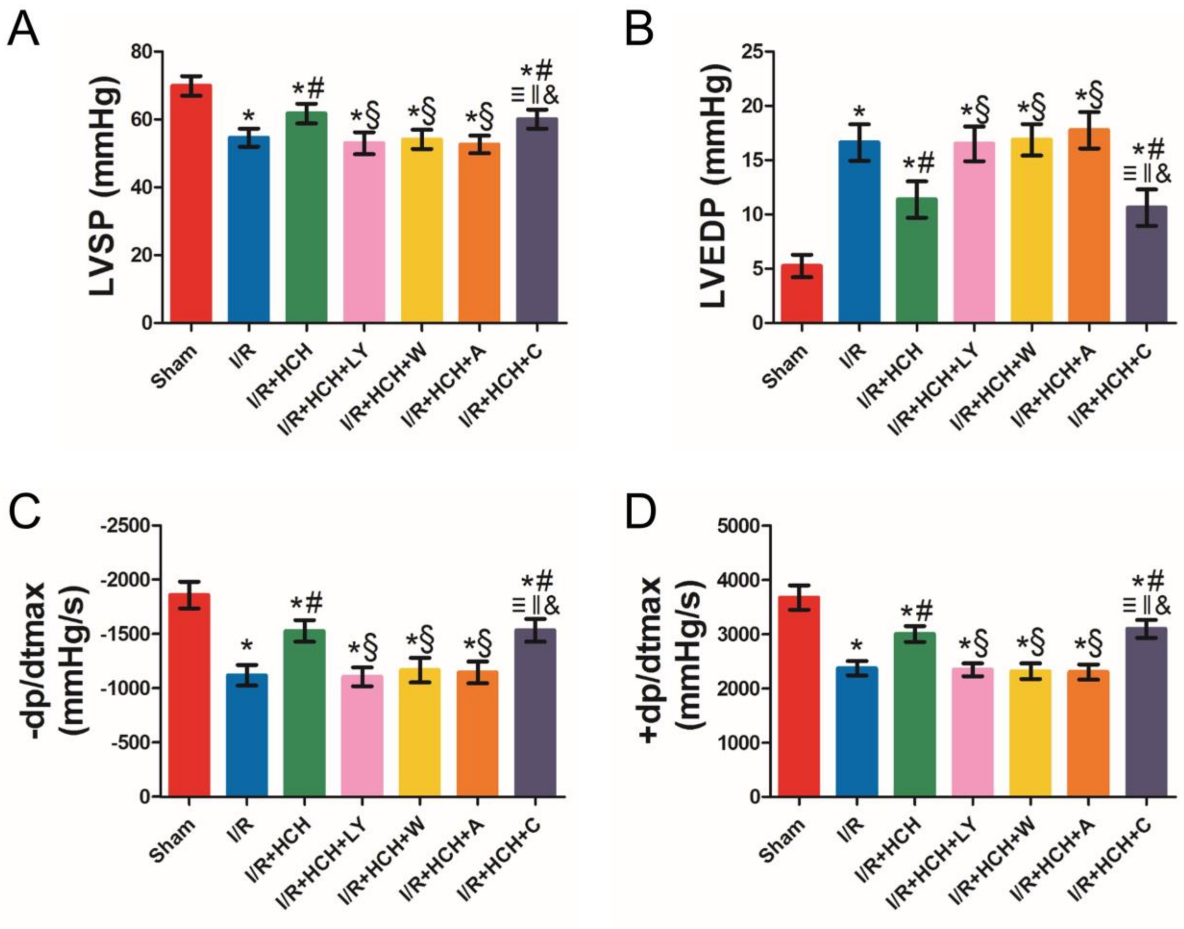

Supplementary Figure 2

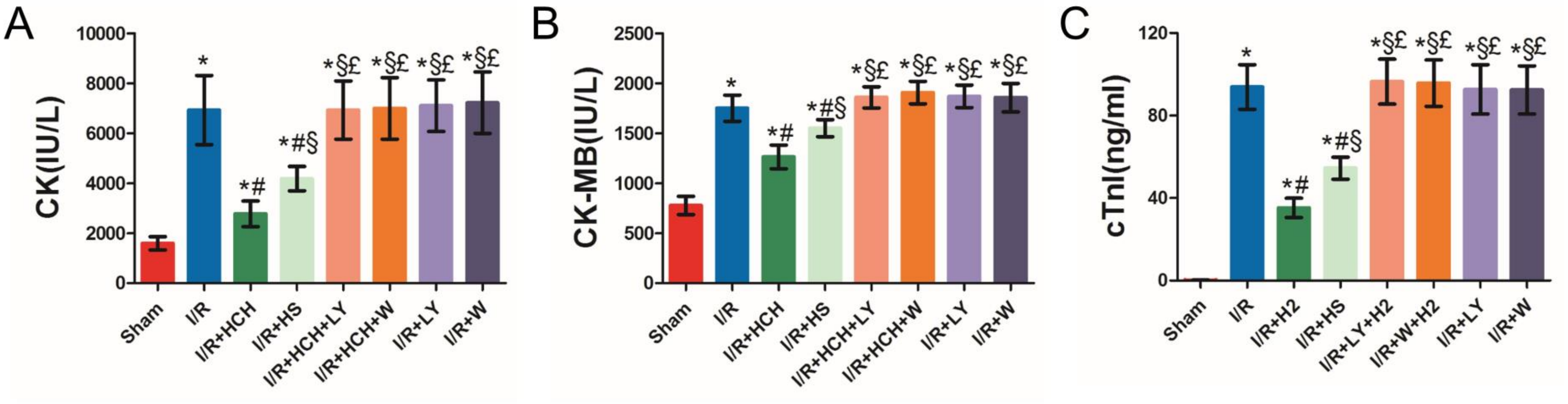

Supplementary Figure 3

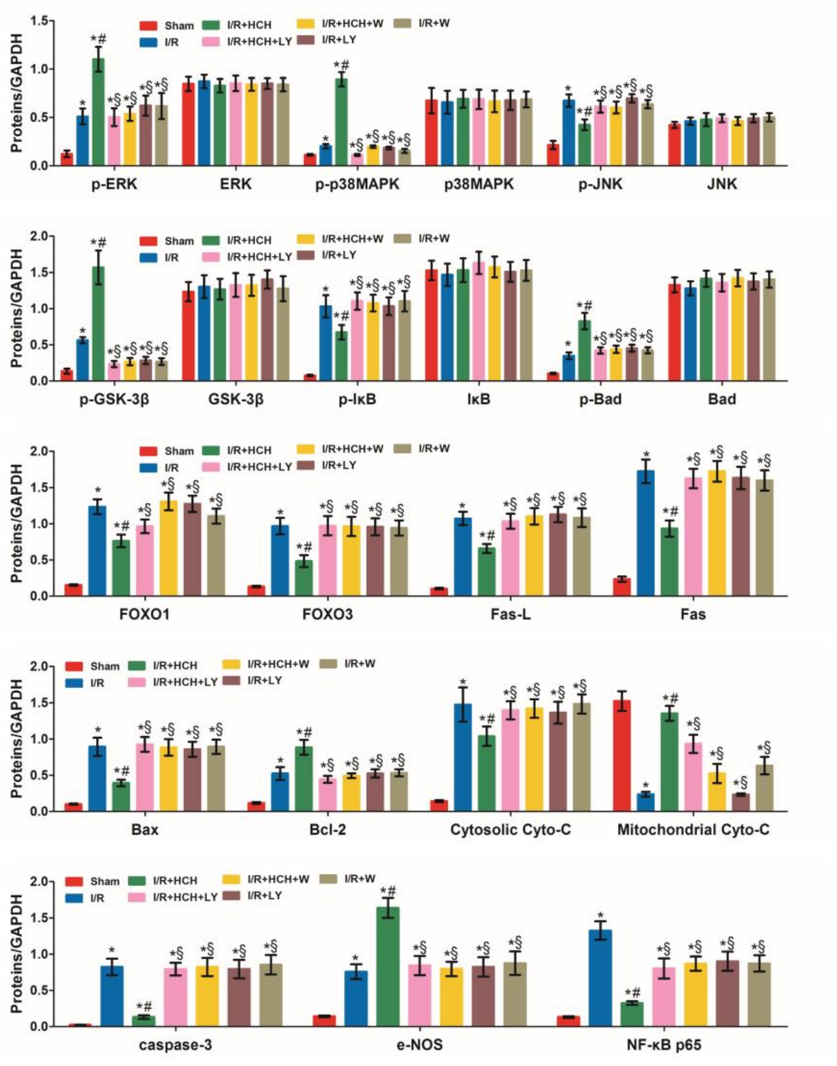

Supplementary Figure 4

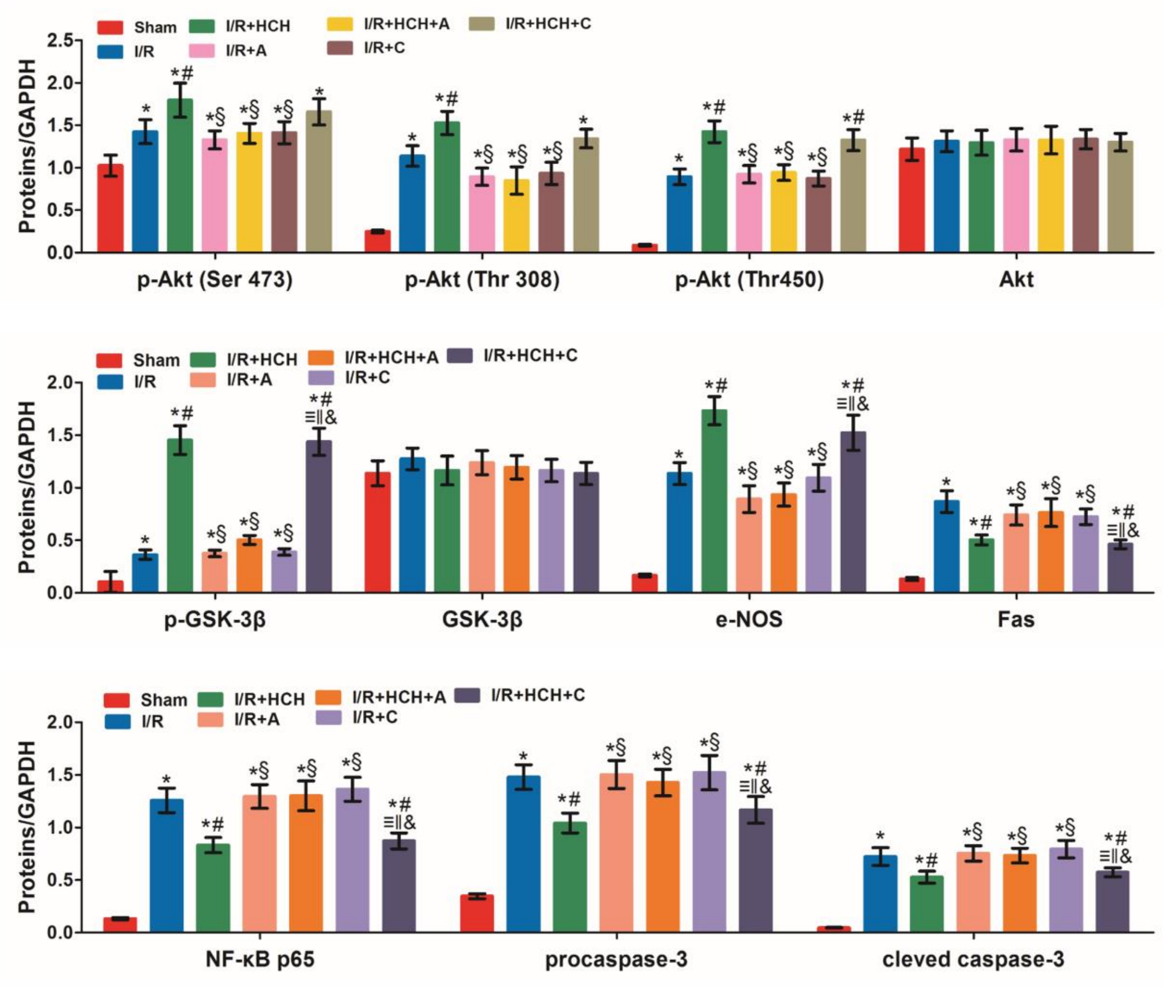

Supplementary Figure 5
